# Supplementary material for: Serological Follow-Up Study Indicates High Seasonal Coronavirus Infection and Reinfection Rates in Early Childhood
Source: Microbiol Spectr. 2022 Jun 21;10(3):e01967-21. doi: 10.1128/spectrum.01967-21 (PMC9241850; doi:10.1128/spectrum.01967-21)
Supplement: SUPPLEMENTAL FILE 1 — Supplemental material. Download spectrum.01967-21-s0001.pdf, PDF file, 1.1 MB [file spectrum.01967-21-s0001.pdf]

Serological follow-up study indicates high seasonal coronavirus infection and reinfection rates in early childhood

By Kolehmainen et. al

Supplementary Figures 1-4

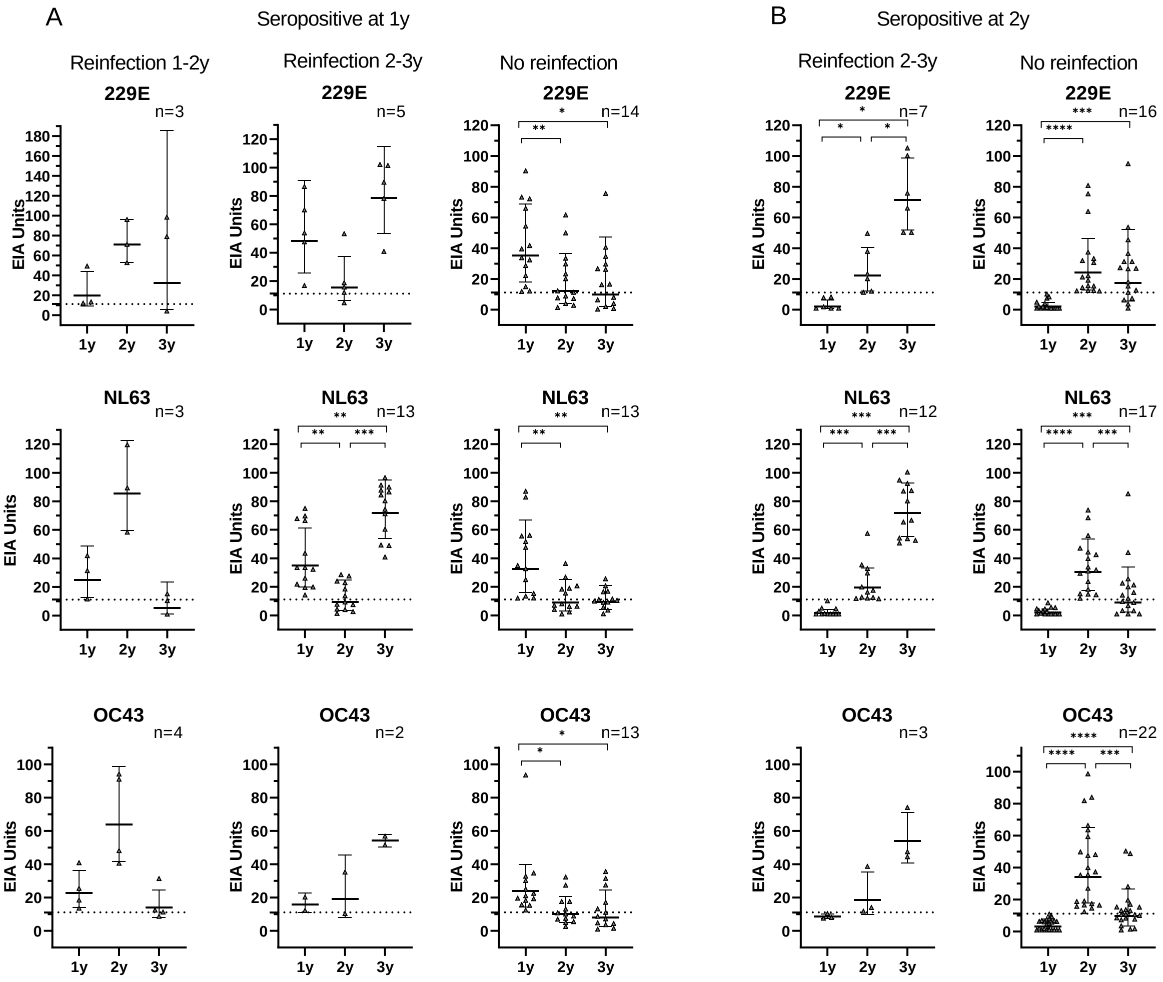

Supplementary figure 1. Antibody levels in HCoV-seropositive children with or without reinfection. IgG antibody levels are shown for children who were first seropositive for an HCoV at 1 year (A) or 2 years (B). The children were divided in groups based on the timing they showed an increase of >20 EIA units between adjacent samples in antibody levels or if they showed no increase in antibody levels. The number of children in each group (n) is indicated in the graph. Statistical analysis for differences in IgG levels between different age groups were calculated using Wilcoxon matched pairs signed-rank test for groups consisting of >4 samples. Two-tailed p-values *<0.05, **<0.01, ***<0.001 ****<0.0001 were considered significant. Geometric means and standard deviations are shown in the figure. Dashed line indicates the assay cut-offs.

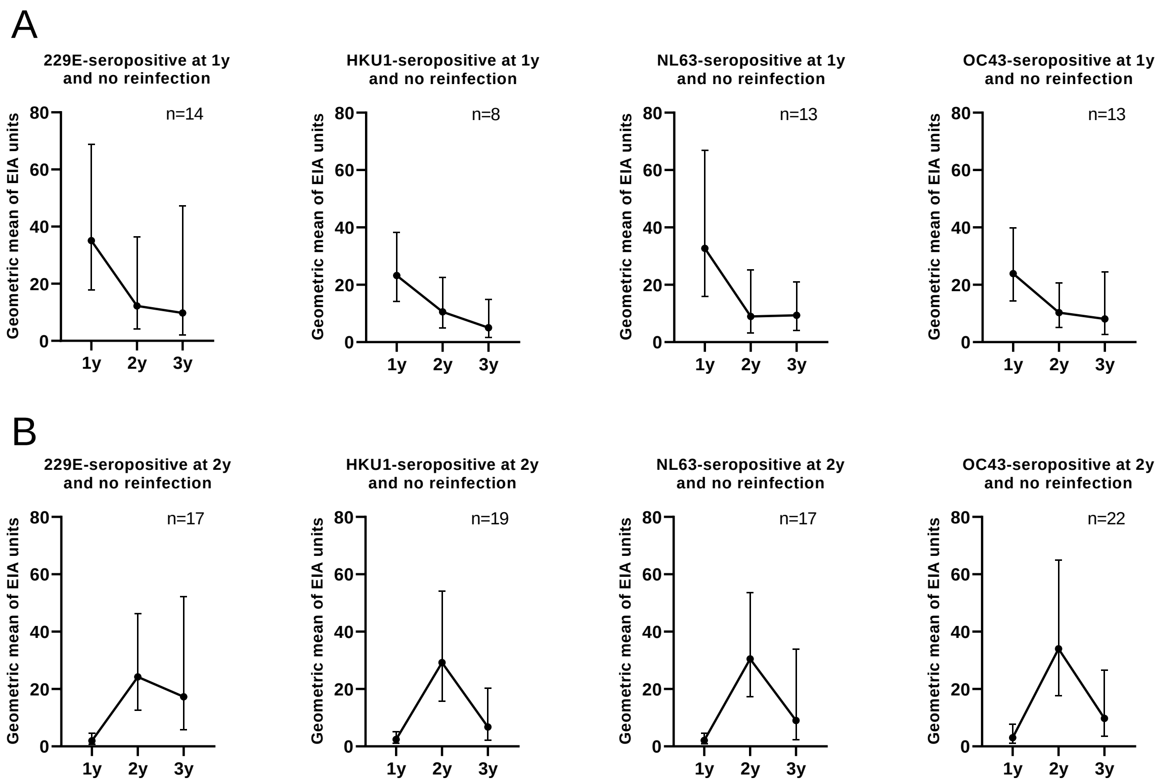

Supplementary figure 2. Kinetics of the decline of anti-HCoV IgG level after primary infection. Geometric means of anti-HCoV IgG antibody levels from groups of children who had no evidence of reinfection and who had contracted their primary infection by the age of 1 year (A) or 2 years (B) were calculated at time points of 1, 2 and 3 years. The geometric means and standard deviations of geometric mean IgG antibody levels at each time point and the number of children in each group (n) are shown in the figure.

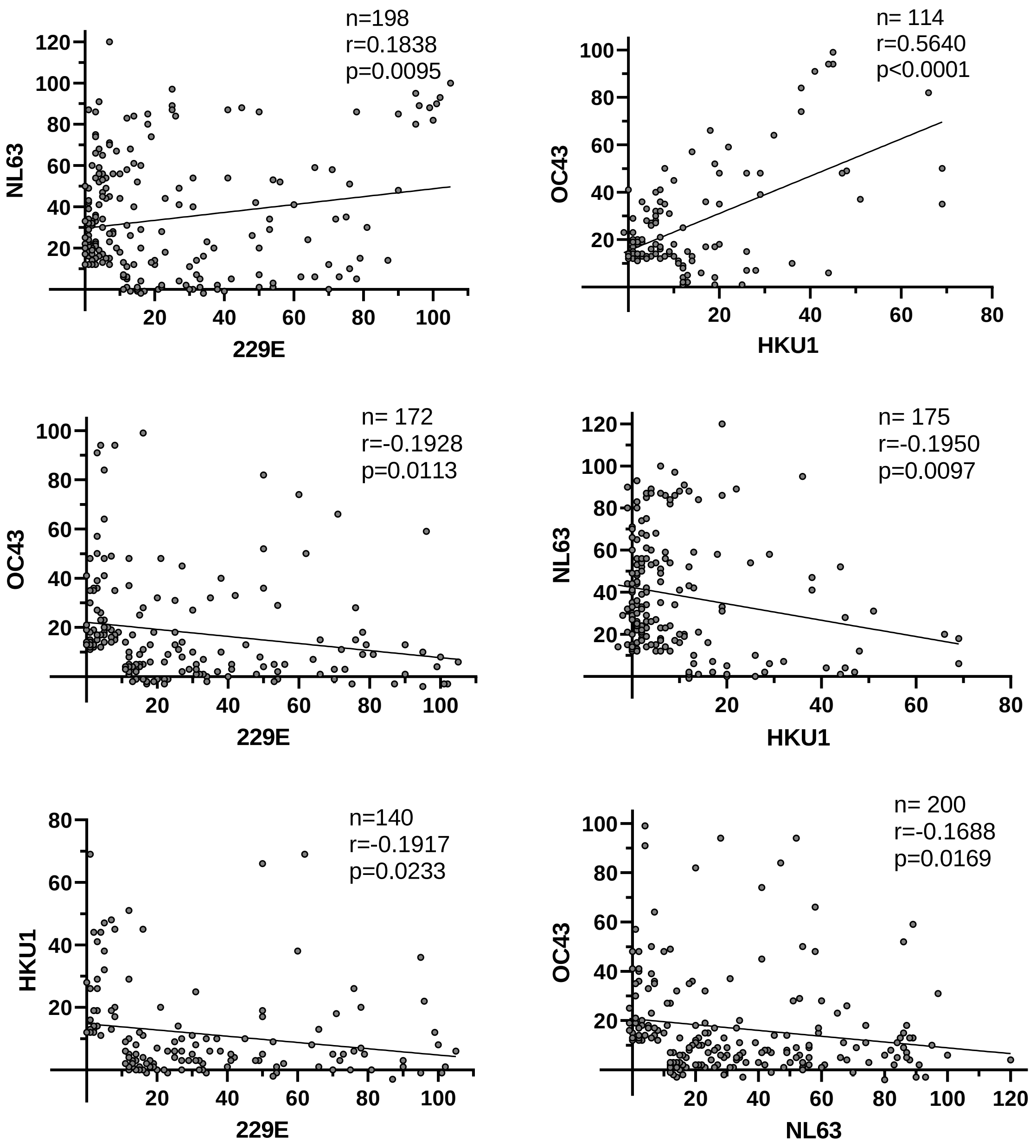

Supplementary figure 3. Correlation of anti-HCoV N IgG antibody levels between different HCoV types for samples that were positive for at least one of the HCoVs in the correlation. Correlation coefficients of IgG antibody levels for anti-HCoV N assays were determined with Pearson's correlation test. Sample numbers (n), correlation test coefficients (r), p values, and regression lines are shown.

A

	SARS-2	MERS	HKU1	OC43	229E	NL63
SARS-2						
MERS	50 %					
HKU1	32 %	32 %				
OC43	30 %	32 %	66 %			
229E	25 %	24 %	25 %	24 %		
NL63	24 %	24 %	22 %	22 %	48 %	

B

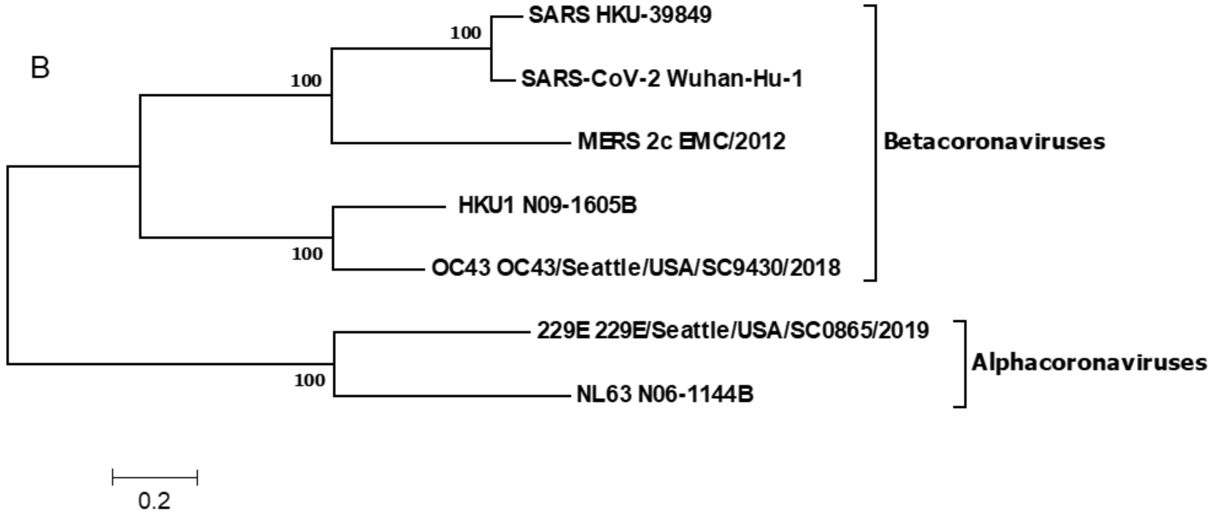

Supplementary figure 4. Human coronavirus N similarities and phylogenetic relationships. ClustalW-aligned and codon-based HCoV N sequences were used to compare amino acid sequence identities (A) and the nucleotide sequences were used to estimate phylogenetic relationships (B). Maximum likelihood method with a general time-reversible algorithm and a gamma distribution correction were used to analyze the phylogenetic relationship. The phylogenetic tree was confirmed with 100 bootstrap replicates. The name of the virus is shown in the figure and the GenBank accession numbers for the N protein sequences are indicated in Materials and Methods.
